# PRMT5 Mediates Sepsis‐Associated Lung Injury by Modulating JAK1 Arginine Methylation: A Mechanism Study

**DOI:** 10.1002/kjm2.70174

**Published:** 2026-01-22

**Authors:** Bo Wang, Zhen Ge, Fei‐Xiang Chen

**Affiliations:** ^1^ Department of Rehabilitation Medicine No. 903 Hospital of PLA Joint Logistic Support Force Hangzhou Zhejiang China

**Keywords:** arginine methylation, JAK1/STAT3, PRMT5, sepsis‐associated lung injury

## Abstract

Lung injury is a common complication in critical sepsis. PRMT5 is implicated in endothelial inflammation and lung diseases, but its role in sepsis‐associated lung injury remains unclear. This study collected clinical sepsis samples and detected the mRNA expression of PRMT5. Subsequently, a murine sepsis model (CLP) was constructed to assess disease severity (survival, sepsis score, temperature, weight). Then, lung histopathology was evaluated with HE staining. ELISA evaluated the expression of inflammatory cytokines in mice blood, and immunohistochemistry detected PRMT5 expression. In vitro, a sepsis cell model was generated by LPS stimulation of human pulmonary microvascular endothelial cells (HPMECs). qRT‐PCR confirmed transfection efficiency. CCK‐8 assay, ELISA, MDA/T‐AOC kits, and flow cytometry tested cell viability, inflammatory cytokines, oxidative stress markers, and apoptosis, respectively. Bioinformatic analysis predicted PRMT5‐interacting proteins, validated by Co‐IP and immunofluorescence. JAK1 arginine methylation, JAK1 protein stability, and activation of the JAK1/STAT3 pathway were assessed by Western blot. The results showed that PRMT5 was upregulated in sepsis patients. PRMT5 knockdown attenuated septic symptoms in CLP mice, manifested by increased survival, reduced sepsis scores, restored physiological parameters, and alleviated lung injury. PRMT5 silencing reversed LPS‐induced decreased viability of HPMECs, inflammatory cytokine release, and oxidative product accumulation. Mechanistically, PRMT5 stabilizes JAK1 protein through arginine methylation, activates the JAK1/STAT3 signaling pathway, and thereby promotes inflammatory responses and oxidative damage. In summary, PRMT5 regulates sepsis‐induced lung injury through a methylation‐dependent JAK1/STAT3 pathway, serving as a potential target for clinical intervention.

## 1 Introduction

Sepsis is a life‐threatening clinical syndrome triggered by infection, hallmarked by a dysregulated host response that culminates in organ dysfunction, typified by systemic inflammation and concomitant immunosuppression [1, 2]. Among all organs, the lung is the earliest and most frequently injured during sepsis [3], and sepsis‐associated lung injury (SALI) has become the primary cause of death in patients with sepsis [4]. SALI is characterized by diffuse alveolar damage, endothelial dysfunction, and localized inflammatory responses [5]. Without prompt diagnosis and early intervention, SALI can rapidly evolve into severe respiratory failure, with mortality rates reaching 40% [6]. Current management of SALI remains largely supportive, relying on fluid resuscitation, vasoactive agents, and mechanical ventilation [7]. These measures, however, fail to interrupt the underlying pathological cascade, resulting in limited therapeutic efficacy [8]. Thus, elucidating the molecular mechanisms driving SALI and translating these insights into targeted therapies are urgently needed.

Accumulating evidence has implicated posttranslational modifications (PTMs) in the initiation and progression of SALI [9, 10]. PTMs refer to enzymatically catalyzed chemical alterations at specific amino‐acid residues after protein synthesis, including phosphorylation, acetylation, ubiquitination, and methylation [11]. By modulating intracellular signaling, protein stability, subcellular localization, and protein–protein interaction networks, PTMs endow proteins with functional diversity [12]. Among these modifications, arginine methylation, catalyzed by protein arginine methyltransferases (PRMTs), transfers methyl groups to arginine residues within target proteins [13]. As an important PTM, it critically regulates pathophysiological processes such as cancer [14], inflammatory responses [15], and immune modulation [16]. For instance, PRMT7‐mediated arginine methylation promotes inflammatory lung injury by enhancing monocyte recruitment to the lung [17]. Nevertheless, the roles of PRMT family members and their arginine methylation events in SALI remain largely unknown.

PRMT5, a well‐characterized type II PRMT, catalyzes symmetric dimethylation of histone and nonhistone substrates, governing diverse cellular processes including signal transduction, DNA‐damage responses, gene regulation, and RNA splicing [18, 19]. Aberrant PRMT5 expression is closely linked to inflammatory disorders. Elevated PRMT5 levels exacerbate pulmonary inflammation and tissue remodeling in murine models [20], whereas PRMT5 knockdown mitigates oxidized low‐density lipoprotein‐induced oxidative stress in endothelial cells [21]. PRMT5 participates in the regulation of inflammatory and oxidative pathways. However, its precise molecular mechanisms and translational potential in SALI have yet to be defined.

This study first observed significant PRMT5 upregulation in clinical samples from patients with sepsis. Using in vivo and in vitro sepsis models, we demonstrated that PRMT5 knockdown attenuated histopathological lung injury in septic mice and suppressed lipopolysaccharide (LPS)‐induced inflammatory responses and oxidative stress in human pulmonary microvascular endothelial cells (HPMECs). PRMT5 bound to JAK1 and catalyzed its arginine methylation, thereby activating the JAK1/STAT3 signaling axis and ultimately amplifying sepsis‐associated inflammatory cascades and oxidative damage. These findings established PRMT5 as a critical modulator of SALI and provided a theoretical framework for developing PRMT5‐targeted therapeutic strategies.

## 2 Materials and Methods

### 2.1 Bioinformatics

Potential interactors of PRMT5 were queried in the BioGRID database (https://thebiogrid.org/). Arginine methylation sites within the JAK1 protein were predicted on the online tool PRmePRed (https://bioinfo.icgeb.res.in/PRmePRed/).

### 2.2 Clinical Samples

We recruited 20 subjects at No. 903 Hospital of PLA Joint Logistic Support Force from January 2025 to May 2025, including 10 patients with sepsis (clinical information presented in Supporting Information Material 1) and 10 healthy controls. Inclusion criteria strictly followed the Sepsis 3.0 definitions published in the Journal of the American Medical Association, 2016: (1) age ≥ 18 years; (2) sample collected within 24 h of admission; (3) no prior medication before sampling. Exclusion criteria were: (1) pregnancy; (2) age < 18 years; (3) autoimmune disease; (4) history of thoracic trauma or cardiac surgery; (5) organic heart disease (e.g., coronary artery disease, cardiomyopathy, acute cor pulmonale) or congenital heart disease; (6) chronic renal insufficiency or neuromuscular disorders; (7) malignancy; (8) medication prior to sampling; (9) incomplete clinical data or readmission; and (10) voluntary withdrawal during treatment. Peripheral venous blood was collected from all participants. Serum was separated by centrifugation and stored at −80°C until use. The study protocol was approved by the Medical Ethics Committee of No. 903 Hospital of PLA Joint Logistic Support Force (Approved No. 20240719/03/01/008), and written informed consent was obtained from all participants.

### 2.3 qRT‐PCR

Total RNA was isolated with TRIzol reagent (Thermo Fisher Scientific, USA), followed by reverse transcription employing the PrimeScript RT Kit (Takara, Japan). Subsequent quantitative PCR reactions were carried out on an ABI 7500 instrument (USA) in conjunction with SYBR Premix Ex Taq II (Takara, Japan). All reactions were executed in triplicate. GAPDH functioned as the internal reference, and target‐gene abundance was determined by 2^−ΔΔ*C*^_t_ and reported as fold change. Primer sequences are listed in Table 1.

**TABLE 1:** Primer sequences.

Gene	Forward primer (5′ → 3′)	Reverse primer (5′ → 3′)
PRMT5	CTGACACACTAGGGGCTGTG	ACTAGTCTGCCCTTCTCCGT
GAPDH	AATGGGCAGCCGTTAGGAAA	GCGCCCAATACGACCAAATC

### 2.4 Experimental Animals

Eighteen male C57BL/6J mice aged 9–10 weeks and weighing 25–29 g were purchased from Hunan SJA Laboratory Animal Co. Ltd. (China). Animals were housed under specific‐pathogen‐free conditions at 60%–65% humidity and 22°C–25°C with a 12/12 h light/dark cycle, and provided ad libitum access to food and water. Experiments began after a 1‐week acclimation period and only healthy mice were used. All procedures were approved by the Animal Experimentation and Welfare Ethics Committee of Zhejiang Luoxi Medical Technology Co. Ltd., Hangzhou, China (Approved No. LX4825010601) and conducted in accordance with the Guide for the Care and Use of Laboratory Animals issued by the Ministry of Science and Technology of the People's Republic of China.

### 2.5 Animal Model Construction

Mice were randomly allocated into two groups: sham (*n* = 6) and cecal ligation and puncture (CLP, *n* = 12). After skin disinfection, mice were anesthetized with 1%–2.5% isoflurane inhalation and a 1 cm midline abdominal incision was made to exteriorize the cecum. A 3–0 silk ligature was placed at the mid‐portion of the cecum, which was punctured twice with a 21 gauge needle approximately 0.5 cm away from the blind end. Gentle pressure was applied to extrude a small amount of feces to ensure patency. The cecum was returned to the abdomen and the incision was closed. Sham‐operated mice underwent identical procedures without ligation or puncture. Post‐operatively, all mice received 1 mL of 37°C normal saline subcutaneously for fluid resuscitation.

CLP mice were further divided into CLP + sh‐NC (*n* = 6) and CLP + sh‐PRMT5 (*n* = 6) groups. Twenty‐four hours before CLP, mice in the sh‐NC group received 10 μg of adeno‐associated virus (AAV9‐sh‐NC, Vigene Biosciences, China) via tail‐vein injection, whereas mice in the sh‐PRMT5 group received 10 μg of AAV9‐sh‐PRMT5. Survival was monitored daily for 3 days post‐operation. At the end of the experiment, all mice were euthanized. Blood and lung tissues were collected for subsequent analyses and histological examination.

### 2.6 Survival Analysis and Lung Injury Scoring

Kaplan–Meier survival curves were plotted and analyzed using GraphPad Prism 8.0 (GraphPad, USA). Lung injury was graded by a pathologist blinded to group assignment based on the degree of inflammatory cell infiltration, interstitial thickening, edema, and hemorrhage. Each parameter was scored from 0 (normal) to 4 (severe).

### 2.7 Physiological Indices

Rectal temperature was measured at 0, 2, 4, 6, and 8 h postinfection using a digital rectal thermometer (Beijing Jianyi Technology Development Co. Ltd., China). Body weight was recorded on days 0, 7, 14, 21, and 28 postinfection.

### 2.8 HE Staining

Five micrometers thick lung sections were fixed in 10% neutral‐buffered formalin (Biosharp, China) at 4°C overnight, dehydrated through graded ethanol, cleared and embedded in paraffin. Sections were stained with hematoxylin (Beyotime, China) for 10 min and eosin (Beyotime, China) for 5 min, then examined under a microscope (Carl Zeiss, Germany).

### 2.9 ELISA

Serum and cell‐culture supernatant levels of TNF‐α, IL‐1β, and IL‐6 were quantified using ELISA kits (Elabscience, China). Briefly, 0.1 mL sample was added to 96‐well plates and incubated at 37°C for 1 h. After washing, 0.1 mL freshly diluted enzyme‐conjugated antibody was added and incubated for 0.5–1 h at 37°C. Then, 0.1 mL TMB substrate was added and incubated for 10–30 min at 37°C. The reaction was stopped by adding 0.05 mL of 2 M sulfuric acid to each well. Optical density was read at 450 nm with a microplate reader (Thermo Fisher Scientific, USA), and cytokine concentrations were calculated.

### 2.10 Immunohistochemistry (IHC) Staining

Sections were immersed in 0.01 M citrate buffer (pH 6.0) (Coolaber, China), heated in a microwave until boiling, then maintained at boiling for 20 min. After natural cooling, slides were brought to room temperature and rinsed three times with 0.01 M PBS (3 min each). Endogenous peroxidase activity was blocked with 1% periodic acid for 10 min at room temperature, followed by three PBS washes of 3 min each. Sections were incubated with anti‐PRMT5 antibody (ab109451, Abcam, UK) overnight at 4°C. The next day, 100 μL Goat anti‐Rabbit IgG H&L (HRP) (ab6721, Abcam, UK) was applied for 30 min at 37°C, followed by 100 μL DAB (Yeasen, China) for 5 min at room temperature for color development. Nuclei were counterstained with hematoxylin (Beyotime, China) for 10 min and rinsed with distilled water. After sequential dehydration in graded ethanol (60% to 100%, 5 min each) and xylene clearing, slides were mounted with neutral resin and examined under a microscope (Carl Zeiss, Germany).

### 2.11 Cell Culture and LPS Treatment

HPMECs (PRI‐H‐00006) were purchased from Shanghai Zhong Qiao Xin Zhou Biotechnology Co. Ltd. (China) and cultured at 37°C with 5% CO_2_ in complete endothelial cell medium ECM (1001‐b, Zhong Qiao Xin Zhou, China) containing 500 mL ECM basal medium, 25 mL fetal bovine serum, 5 mL endothelial cell growth supplement, and 5 mL penicillin–streptomycin. For the cellular model, 100 ng/mL LPS (Yeasen, China) was added to the conditioned supernatants and incubated for 4 h to induce inflammation. LPS solutions were freshly prepared in sterile PBS and mixed thoroughly before use.

### 2.12 Cell Transfection

Specific expression vectors (oe‐PRMT5, PRMT5‐shRNA, JAK1‐shRNA) and non‐targeting controls were synthesized and purchased from RiboBio (China). pcDNA3.1(+)‐3 × Flag‐PRMT5, pcDNA3.1‐HA‐JAK1 and corresponding empty vectors were obtained from Addgene (USA). HPMECs were seeded in culture dishes and pre‐incubated with serum‐free Opti‐MEM (Gibco, USA) for 1 h before transfection. When confluency reached 70%–80%, Lipofectamine 3000 (Thermo Fisher Scientific, USA) was mixed with plasmid or shRNA, incubated at room temperature for 20 min, and then added to cells. After 48 h at 37°C and 5% CO_2_, cells were harvested for downstream assays.

### 2.13 CCK‐8 Assay

Cell viability was assessed by CCK‐8. HPMECs were plated at 2000 cells/well in 96‐well plates and cultured for 0, 24, 48, 72, or 96 h. Twenty microliters of CCK‐8 reagent (5 mg/mL, Sigma‐Aldrich, USA) was added to each well and incubated for 4 h. Absorbance at 450 nm was measured with a microplate reader (Thermo Fisher Scientific, USA) to observe cell viability.

### 2.14 MDA and T‐AOC Assays

After PBS washing and trypsinization, cells were processed according to the manufacturer's instructions. MDA levels were determined using a Colorimetric MDA Assay Kit (Elabscience, China) and read at 532 nm on a microplate reader (Thermo Fisher Scientific, USA). T‐AOC was measured with a T‐AOC colorimetric kit (Elabscience, China) at 520 nm using the same microplate reader.

### 2.15 Reactive Oxygen Species (ROS) Detection

Intracellular ROS were measured with a DCFH‐DA ROS Assay Kit (Beyotime, China). Cells were incubated with 10 μM DCFH‐DA at 37°C for 30 min in the dark, mixing every 3 to 5 min. After centrifugation (800 rpm, 5 min) and three PBS washes, ROS levels were analyzed by flow cytometry (Beckman, USA).

### 2.16 Western Blot (WB)

Cells were lysed in RIPA buffer (Beyotime, China) containing 1% PMSF (Beyotime, China). Protein concentrations were determined with a BCA Assay Kit (Thermo Fisher Scientific, USA). After separation by SDS‐PAGE, proteins were transferred onto PVDF membranes (Sigma‐Aldrich, USA). Membranes were blocked with 5% BSA (Yeasen, China) for 1 h at room temperature, incubated with primary antibodies overnight at 4°C, then with Goat anti‐Rabbit IgG H&L (HRP) (ab6721, Abcam, UK) for 2 h. Bands were visualized with ECL reagent (Beyotime, China). Relative expression of target proteins was calculated with GAPDH serving as the internal control. Antibody details are provided in Table 2.

**TABLE 2:** Antibody information for WB.

Antibody	Provider (country)	Serial number
Rabbit anti‐PRMT5	Abcam (UK)	ab109451
Rabbit anti‐HA	Abcam (UK)	ab236632
Rabbit anti‐flag	Abcam (UK)	ab205606
Rabbit anti‐ADMA	Cell Signaling Technology (USA)	#13522
Rabbit anti‐p‐STAT3	Abcam (UK)	ab32143
Rabbit anti‐STAT3	Abcam (UK)	ab68153
Mouse anti‐JAK1	Proteintech (China)	66466‐1‐lg
Rabbit anti‐ubiquitin	Proteintech (China)	10201‐2‐AP
Rabbit anti‐GAPDH	Abcam (UK)	ab181602
Goat anti‐Mouse IgG H&L (HRP)	Abcam (UK)	ab205719
Goat anti‐Rabbit IgG H&L (HRP)	Abcam (UK)	ab6721

### 2.17 Co‐Immunoprecipitation (Co‐IP)

Cells from different treatment groups were lysed in RIPA buffer (Beyotime, China) containing 1% PMSF (Beyotime, China). After centrifugation, supernatants were collected. For endogenous IP, supernatants were precleared with Protein A/G agarose beads (Santa Cruz Biotechnology, USA) at 4°C for 30 min. Specific antibodies were pre‐incubated for 10 min in PBS containing 0.1% Triton X‐100 (Beyotime, China) and PMSF, then incubated with the Protein A/G beads (Santa Cruz Biotechnology, USA) at 4°C for 3 h. After washing, proteins were eluted by heating at 100°C for 10 min and analyzed by WB. For exogenous IP, supernatants were incubated overnight at 4°C with anti‐Flag M2 magnetic beads (Sigma‐Aldrich, USA) or anti‐HA magnetic beads (Thermo Fisher Scientific, USA). Beads were washed and eluted twice with PBS containing 200 ng 3 × Flag peptide (Sigma‐Aldrich, USA) or 3 × HA peptide (MCE, USA). Eluted proteins were subjected to WB. IgG antibody (ab172730, Abcam, UK) served as a negative control. Additional antibodies are listed in Table 2.

### 2.18 Immunofluorescence (IF) Colocalization

Cells seeded on round coverslips were fixed with 4% paraformaldehyde (Beyotime, China) for 10 min, permeabilized with 0.1% Triton X‐100 in PBS for 5 min, and blocked with 2% BSA (Yeasen, China) for 60 min at room temperature to inhibit non‐specific binding. Cells were then incubated overnight at 4°C with Rabbit anti‐PRMT5 (ab109451, Abcam, UK) and Mouse anti‐JAK1 (66466‐1‐lg, Proteintech, China). After PBS washes, coverslips were incubated for 2 h at 37°C in the dark with Goat anti‐Rabbit IgG H&L (FITC) (ab6717, Abcam, UK) or Goat anti‐Mouse IgG H&L (Alexa Fluor 555) (ab150118, Abcam, UK). Nuclei were counterstained with DAPI (Beyotime, China) for 5 min at room temperature. Coverslips were mounted with anti‐fluorescence quenching mounting medium (Beyotime, China) and imaged using a fluorescence microscope (Carl Zeiss, Germany) to observe colocalization of relevant proteins.

### 2.19 Protein Stability Assay

HPMECs transfected with sh‐NC or sh‐PRMT5 were seeded in six‐well plates. When confluency reached approximately 70%, cells were treated with 100 μg/mL cycloheximide (CHX, Sigma‐Aldrich, USA) to inhibit protein synthesis. Cells were harvested at 0, 3, 6, and 9 h after the addition, lysed with RIPA buffer (Beyotime, China), and analyzed by WB to detect target protein levels.

### 2.20 Statistical Analysis

Data are presented as mean ± SEM from at least three independent experiments. Statistical analyses were performed using GraphPad Prism 8.0 (GraphPad, USA). Inter‐group comparisons were made with unpaired *t* tests. Multiple groups were compared using one‐way ANOVA. Pearson correlation analysis was used to assess relationships between variables. *p* < 0.05 determined statistical significance.

## 3 Results

### 3.1 PRMT5 Is Up‐Regulated in Sepsis, and Knockdown of PRMT5 Attenuates Lung Injury in CLP Mice

**FIGURE 1:**
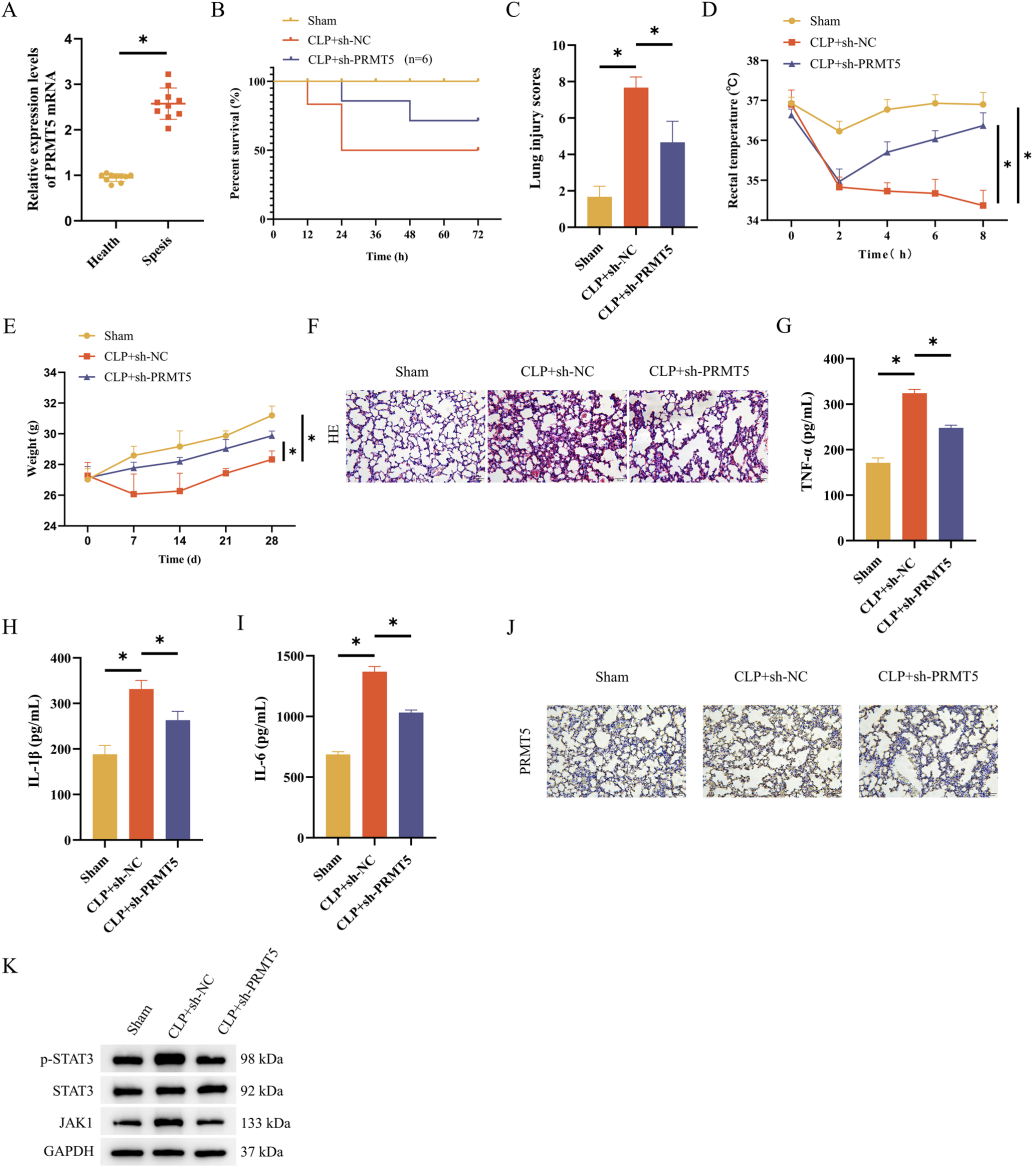
PRMT5 is highly expressed in sepsis, and its knockdown alleviates CLP‐induced lung injury. Blood samples from healthy donors (Health) and patients with sepsis (Sepsis) were collected. (A) The mRNA expression of PRMT5 in the peripheral blood of sepsis patients was increased compared to healthy controls. A sepsis mouse model was established by cecal ligation and puncture (CLP). PRMT5 was knocked down in the model, resulting in three groups: Sham, CLP, and CLP + sh‐PRMT5. (B) Kaplan–Meier survival curves show that PRMT5 knockdown significantly improved survival rate. (C) Lung injury scores indicate that PRMT5 knockdown alleviated lung damage. (D, E) PRMT5 knockdown restored rectal temperature and body weight in CLP mice. (F) HE staining of lung tissue showed pathological changes in each group. PRMT5 knockdown alleviated histopathological lung damage. (G–I) ELISA kits assessed levels of TNF‐α (G), IL‐1β (H), and IL‐6 (I) in the blood of mice in each group. PRMT5 knockdown reduced proinflammatory cytokine release. (J) IHC showed significantly increased PRMT5 expression in CLP mouse lung tissue, which was effectively reduced in the CLP + sh‐PRMT5 group. (K) Western blot indicated that PRMT5 mediated the activation of the JAK1/STAT3 signaling pathway. *p < 0.05.

To determine PRMT5 expression during sepsis and its role in SALI, we first collected blood from 10 volunteers without sepsis and 10 patients with sepsis. qRT‐PCR revealed significantly higher PRMT5 mRNA levels in patients with sepsis than in controls (Figure 1A). We established a CLP mouse model of sepsis and knocked down PRMT5. As displayed in Figure 1B, CLP mice exhibited significantly lower survival than Sham‐operated mice, whereas PRMT5 knockdown improved survival. CLP also increased lung injury severity, an effect that was attenuated by PRMT5 silencing (Figure 1C). Compared with the 0 h baseline rectal temperature of 36.9°C, CLP‐operated mice exhibited a 2.07°C to 2.53°C drop, reaching a nadir of 34.37°C at 8 h postinfection. PRMT5 knockdown partially reversed this hypothermia caused by infection (Figure 1D). PRMT5 knockdown likewise restored body weight in CLP mice, reversing the infection‐induced loss (Figure 1E). Histopathological analysis corroborated these findings. HE staining revealed marked alveolar destruction, hemorrhage, and inflammatory cell infiltration in the lungs of CLP mice, whereas these injuries were substantially attenuated in the CLP + sh‐PRMT5 group (Figure 1F). ELISA demonstrated elevated serum TNF‐α, IL‐1β, and IL‐6 in CLP mice, all of which were significantly decreased following PRMT5 knockdown (Figure 1G–I). IHC confirmed strong PRMT5 immunostaining in CLP lungs that was effectively suppressed in the CLP + sh‐PRMT5 group (Figure 1J). Collectively, these findings demonstrated that PRMT5 was upregulated during sepsis and its silencing attenuated lung injury in CLP mice. To further investigate the effect of PRMT5 knockdown on the activity of the JAK1/STAT3 pathway, we detected the expression of JAK1/STAT3/p‐STAT3 proteins by WB. Compared with the Sham group, the expression of JAK1/p‐STAT3 in the lung tissue of CLP mice was significantly increased, and after knockdown of PRMT5, the expression of JAK1/p‐STAT3 significantly decreased (Figure 1K). This indicated that PRMT5 mediated the activation of the JAK1/STAT3 signaling pathway. The lung protective effect of PRMT5 knockdown is related to the inhibition of the activity of the JAK1/STAT3 pathway.

### 3.2 PRMT5 Knockdown Suppresses LPS‐Induced Inflammatory and Oxidative Injury in HPMECs

**FIGURE 2:**
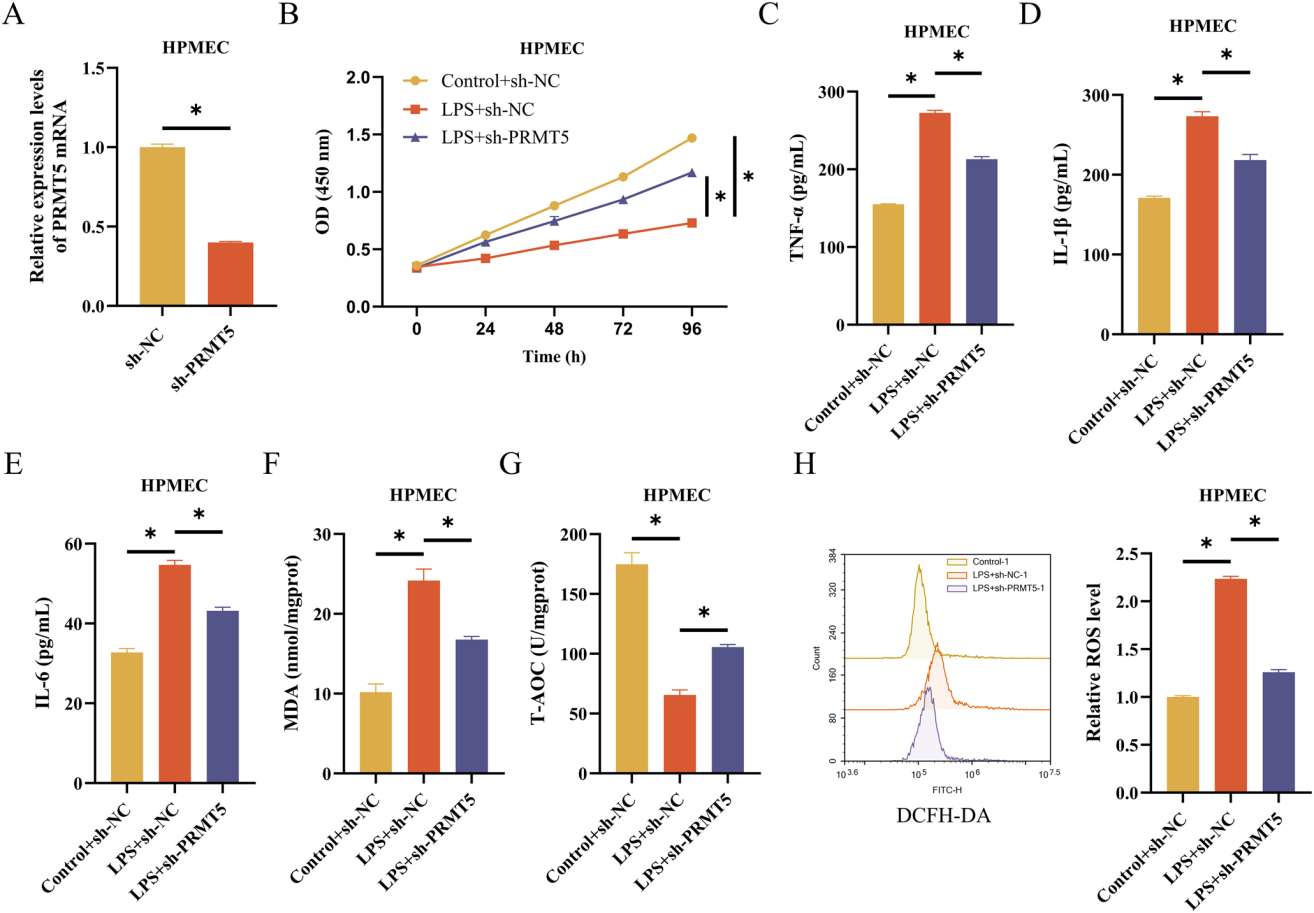
PRMT5 knockdown inhibits LPS‐induced inflammatory and oxidative injury in HPMECs. HPMECs were transfected with sh‐NC or sh‐PRMT5 and treated with LPS (groups: Control+sh‐NC, LPS + sh‐NC, LPS + sh‐PRMT5). (A) Knockdown efficiency was confirmed by qRT‐PCR. (B–H) PRMT5 inhibition exerted protective effects in LPS‐induced inflammatory injury. In HPMECs exposed to LPS, PRMT5 knockdown markedly rescued cell viability (B), suppressed secretion of TNF‐α (C), IL‐1β (D), and IL‐6 (E), reduced lipid peroxidation (MDA, F), enhanced total antioxidant capacity (T‐AOC, G), and lowered intracellular ROS production (H). *p < 0.05.

To clarify the influence of PRMT5 on cellular functions in SALI, HPMECs were transfected with sh‐NC or sh‐PRMT5 and challenged with LPS (groups: Control + sh‐NC, LPS + sh‐NC, LPS + sh‐PRMT5). qRT‐PCR confirmed efficient PRMT5 knockdown by examining its mRNA levels (Figure 2A). CCK‐8 assays showed that LPS significantly reduced the viability of HPMECs, which was reversed by PRMT5 knockdown compared to LPS + sh‐NC (Figure 2B). ELISA revealed that LPS strongly induced TNF‐α, IL‐1β, and IL‐6 secretion, whereas PRMT5 silencing significantly lowered their levels (Figure 2C–E). Oxidative stress analysis demonstrated that LPS elevated MDA content and suppressed T‐AOC activity. Both changes were mitigated by PRMT5 knockdown (Figure 2F,G). Flow cytometry revealed robust ROS accumulation in HPMECs after LPS stimulation, an increase that was attenuated when PRMT5 was silenced (Figure 2H). These findings indicated that PRMT5 knockdown effectively alleviated LPS‐induced inflammatory responses and oxidative damage in HPMECs.

### 3.3 PRMT5 Regulates JAK1 Protein Expression via Arginine Methylation

**FIGURE 3:**
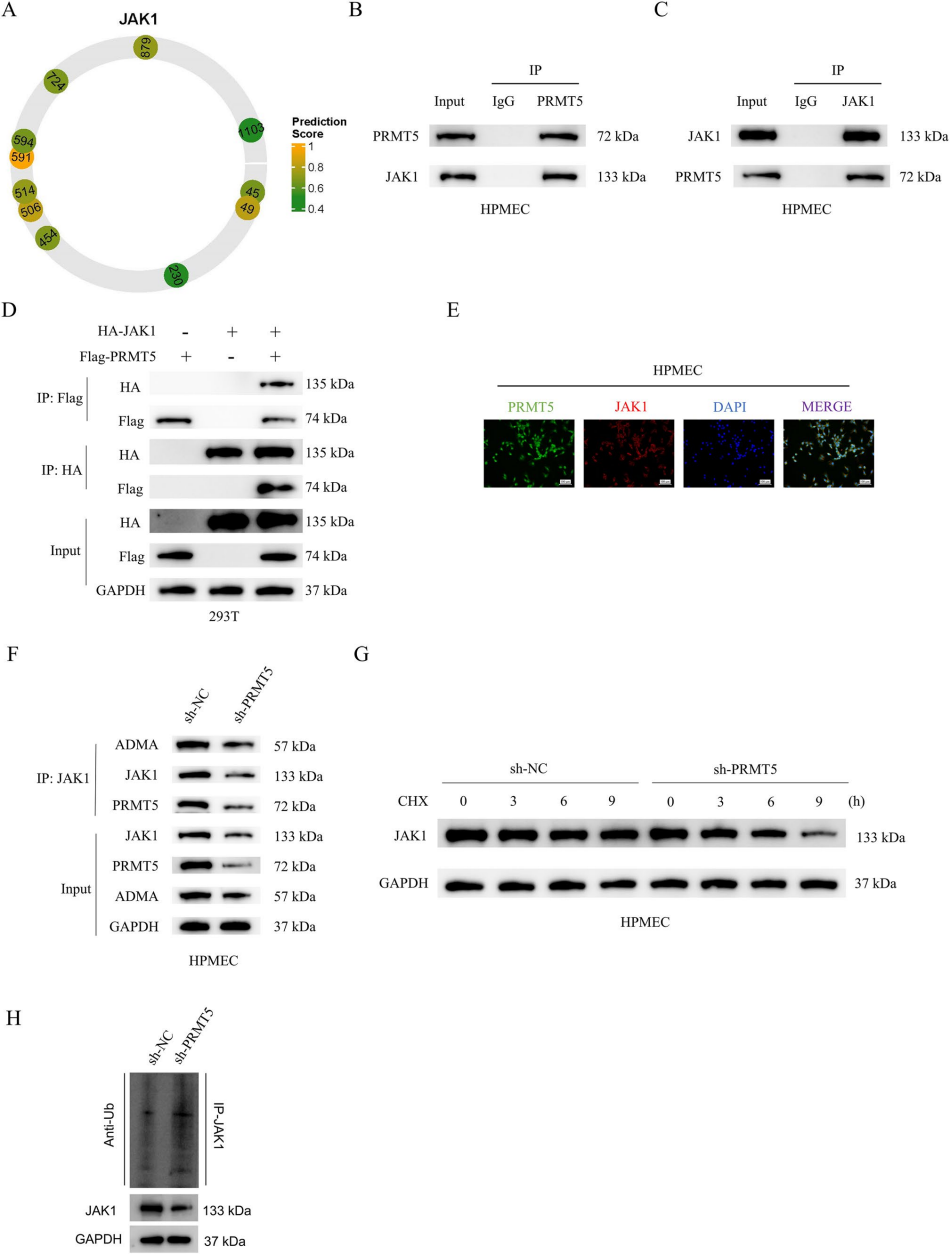
PRMT5 modulates JAK1 expression through arginine methylation. (A) Bioinformatics prediction revealed potential arginine methylation sites in JAK1 (https://bioinfo.icgeb.res.in/PRmePRed/). (B, C) Endogenous Co‐IP confirmed PRMT5‐JAK1 interaction. (D) Exogenous Co‐IP confirmed PRMT5‐JAK1 interaction. (E) Immunofluorescence revealed subcellular colocalization. (F) Co‐IP assays confirmed that PRMT5 knockdown reduced JAK1 methylation. (G) In CHX chase experiments, PRMT5 knockdown accelerated its degradation, suggesting that PRMT5‐mediated methylation stabilized JAK1 protein. (H) Co‐IP assays confirmed that PRMT5 knockdown significantly reinforced the ubiquitination degradation of JAK1.

PRMT5 was initially identified as JAK‐binding protein 1 [19]. The continuous deterioration of sepsis is related to the excessive expression of inflammatory factors induced by the JAK1‐STAT3 signaling pathway. Therefore, we hypothesize that PRMT5 may correlate with JAK1 in sepsis [22]. To clarify the relationship between PRMT5 and JAK1 in SALI, we used the BioGRID database and predicted a potential interaction between PRMT5 and JAK1. Using PRmePRed, we identified multiple putative arginine methylation sites in JAK1 (Figure 3A), suggesting that JAK1 might be a methylation substrate of PRMT5. A Co‐IP experiment was conducted to validate the PRMT5‐JAK1 interaction. First, we used endogenous Co‐IP to detect the interaction between the naturally expressed PRMT5 and JAK1. After precipitation with PRMT5 or JAK1 antibodies, WB detected specific co‐precipitation of endogenous JAK1 or PRMT5, which the IgG isotype control did not show (Figure 3B,C). To rule out antibody‐specific interference, we conducted an exogenous verification. Flag‐PRMT5 and HA‐JAK1 were co‐transfected into HEK293T cells. After precipitation with anti‐Flag antibody, a significant HA‐JAK1 signal was detected, and the reverse Co‐IP (HA antibody precipitation with Flag detection) results were consistent, confirming that the interaction between the two does not depend on the endogenous protein environment (Figure 3D). The above results indicate that PRMT5 physically interacted with JAK1. IF colocalization assays demonstrated prominent cytoplasmic colocalization signals for PRMT5 and JAK1 (Figure 3E). To determine whether PRMT5 methylates JAK1, we performed Co‐IP with a JAK1 antibody in HPMECs transfected with sh‐NC or sh‐PRMT5. Results showed that knockdown of PRMT5 significantly reduced arginine methylation of JAK1 (Figure 3F). CHX chase assays revealed that JAK1 protein degradation was significantly accelerated after PRMT5 knockdown, indicating reduced protein stability (Figure 3G). Furthermore, JAK1 ubiquitination was detected in PRMT5‐knockdown cells through Co‐IP. PRMT5 knockdown significantly enhanced the ubiquitination degradation of JAK1 (Figure 3H), indicating that PRMT5 can inhibit the ubiquitination degradation pathway of JAK1. In summary, PRMT5 bound to JAK1 and stabilized it via arginine methylation, which may be achieved by inhibiting the ubiquitination degradation pathway of JAK1.

### 3.4 PRMT5 Promotes LPS‐Induced Inflammatory and Oxidative Injury in HPMECs by Activating the JAK1/STAT3 Pathway

**FIGURE 4:**
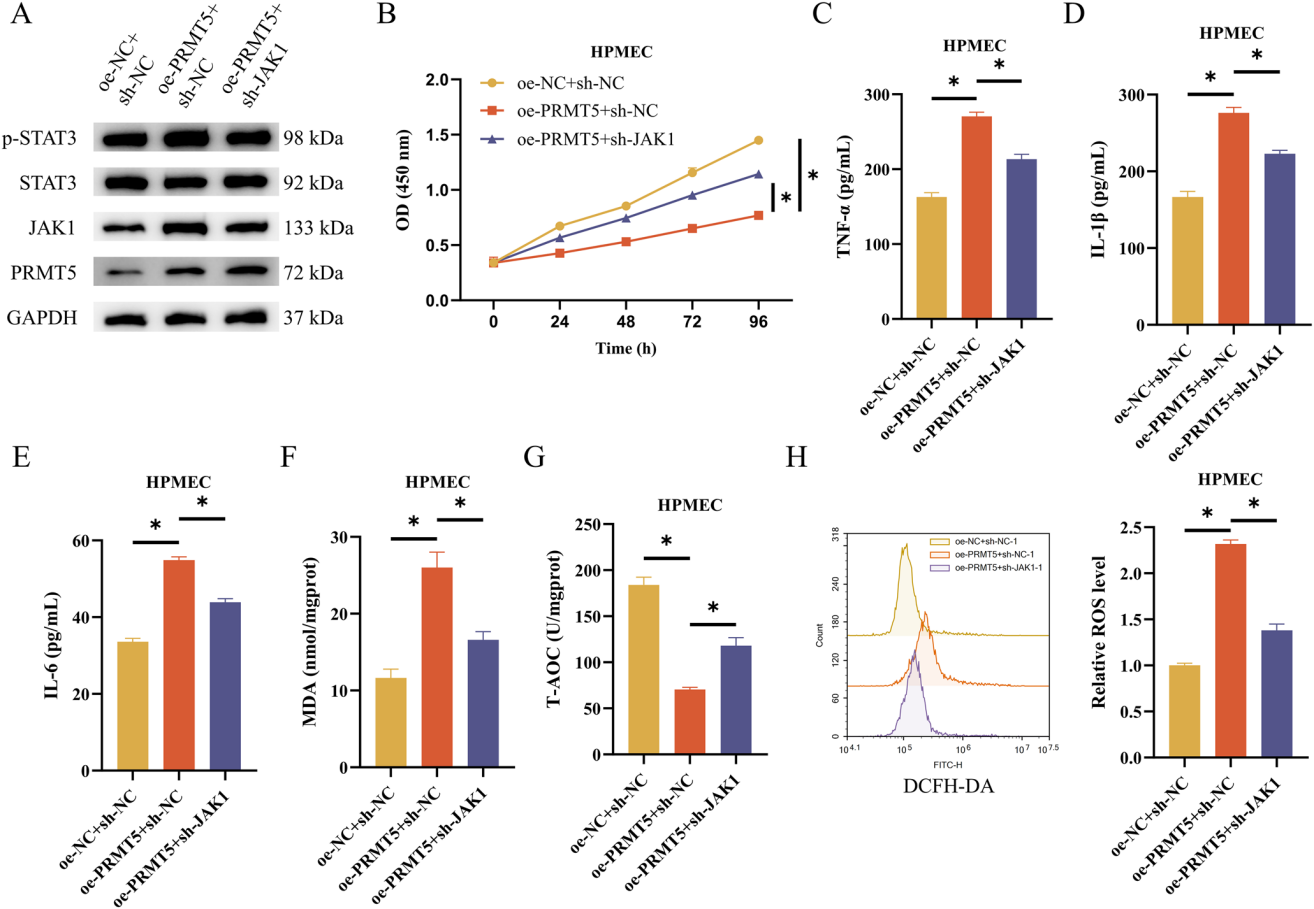
PRMT5 exacerbates LPS‐induced inflammatory and oxidative injury via the JAK1/STAT3 pathway. HPMECs were transfected with oe‐NC + sh‐NC, oe‐PRMT5 + sh‐NC or oe‐PRMT5 + sh‐JAK1 and stimulated with LPS. (A) Western blot showed that PRMT5 overexpression increased the expression level of JAK1 and p‐STAT3, which was reversed by JAK1 silencing. (B–H) PRMT5 promoted inflammatory response and oxidative damage through JAK1/STAT3 signaling. PRMT5 overexpression exacerbated LPS‐induced cytotoxicity (B), cytokine production of TNF‐α (C), IL‐1β (D), and IL‐6 (E), increased the level of lipid peroxidation (MDA, F), reduced total antioxidant capacity (T‐AOC, G), and increased intracellular ROS production (H); co‐knockdown of JAK1 reversed these effects. *p < 0.05.

We investigated whether PRMT5‐mediated arginine methylation of JAK1 contributes to SALI. PRMT5‐overexpression (oe‐PRMT5) or JAK1‐knockdown (sh‐JAK1) model was constructed in HPMECs. LPS was used to induce inflammatory responses. WB revealed that PRMT5 overexpression increased JAK1 and p‐STAT3 protein levels in the oe‐PRMT5 + sh‐NC group, whereas JAK1 knockdown reversed the activation of the JAK1/STAT3 pathway by PRMT5 overexpression (Figure 4A). CCK‐8 assays showed that JAK1 knockdown restored cell viability suppressed by PRMT5 overexpression (Figure 4B). ELISA demonstrated that PRMT5 overexpression elevated TNF‐α, IL‐1β, and IL‐6 secretion, and JAK1 knockdown attenuated this proinflammatory effect (Figure 4C–E). Oxidative stress assays revealed that PRMT5 overexpression increased MDA content and decreased T‐AOC activity, which were reversed by JAK1 knockdown (Figure 4F,G). Flow cytometry further confirmed that ROS accumulation induced by PRMT5 overexpression was mitigated by JAK1 knockdown (Figure 4H). In summary, PRMT5 exacerbated LPS‐induced inflammatory and oxidative injury in HPMECs by activating the JAK1/STAT3 pathway.

## 4 Discussion

Lung injury is the most common organ dysfunction in sepsis, yet improving its clinical prognosis remains a major challenge. Elucidating SALI pathogenesis is therefore essential for developing targeted therapies. We confirmed that PRMT5 enhanced the protein stability of JAK1 via arginine methylation, thereby activating the JAK1/STAT3 signaling axis and promoting inflammatory and oxidative damage in HPMECs. Our findings identified the PRMT5/JAK1/STAT3 pathway as a critical regulatory hub in SALI and provided new mechanistic insights into its development.

PRMT5 is a key arginine methyltransferase in mammalian cells that modulates RNA splicing, cell‐cycle progression, cell death and signal transduction [19]. It has been implicated in cancer [23], cardiovascular disease [24], periodontitis [25] and other disorders. Lim et al. [26] reported that PRMT5 participates in hypoxia‐ and ischemia‐induced apoptosis of lung epithelial cells in vitro and in vivo. PRMT1‐mediated arginine methylation stabilizes EGR1 and triggers acute lung injury [27]. However, the role of PRMT5 in sepsis and SALI remains poorly understood. We observed markedly elevated PRMT5 expression in blood samples from patients with sepsis compared to healthy controls. PRMT5 knockdown improved survival in CLP mice, ameliorated hypothermia and body weight loss, and reduced lung injury, indicating a pathogenic role for PRMT5 in SALI. Ju et al. [28] showed that PRMT5 aggravates cigarette smoke extract‐induced bronchial epithelial inflammation by up‐regulating CXCL10, and Diao et al. [29] demonstrated that PRMT5 inhibition attenuates oxidative stress by activating the Nrf2/HO‐1 pathway. In line with these reports, our in vitro experiments revealed that PRMT5 knockdown effectively counteracted LPS‐induced reductions in HPMEC viability, exaggerated release of inflammatory cytokines and heightened oxidative stress. Nevertheless, the precise molecular mechanism by which PRMT5 drives inflammation and oxidative damage remains incompletely understood.

PRMT5 was initially identified as a JAK binding protein 1 [19]. Bioinformatic analysis in the present study predicted an interaction between PRMT5 and JAK1 and identified potential arginine methylation sites within JAK1. Co‐IP and IF colocalization experiments confirmed their binding. PRMT5 knockdown not only decreased arginine methylation of JAK1 but also weakened the stability of its protein, suggesting that PRMT5 modulated the expression and function of JAK1 via arginine methylation. JAK1, a core member of the JAK family, phosphorylates downstream STATs such as STAT3, initiating JAK1/STAT3 signaling [30]. Activation of the JAK1/STAT3 pathway generates abundant proinflammatory mediators and is critical in sepsis‐driven inflammation and tissue injury [22]. Xu et al. [31] reported that blocking JAK1/STAT3 effectively alleviates LPS‐induced lung injury, thereby attenuating inflammation and apoptosis. Consistently, we found that JAK1 knockdown reversed PRMT5 overexpression‐induced inflammatory and oxidative injury. Collectively, the pivotal role of the PRMT5/JAK1/STAT3 axis in sepsis‐related inflammatory damage provided new insights into the pathogenic mechanism of SALI.

Our clinical analysis revealed significant upregulation of PRMT5 in patients with sepsis and delineated a pathway in which PRMT5 regulated the stability of JAK1 protein through arginine methylation and activated JAK1/STAT3 signaling, thereby contributing to SALI. However, although we confirmed that PRMT5 stabilized JAK1 through arginine methylation, the specific modification sites have not yet been identified. Future research will employ high‐resolution mass spectrometry analysis to precisely locate the methylation modification sites on the JAK1 gene. Targeted mutation experiments will be designed to evaluate the effects of specific methylation events on the transcriptional activity, protein expression, and downstream signaling pathways (such as JAK‐STAT) of JAK1. Nonetheless, this study expanded current understanding of PRMT5 in SALI and provided a theoretical basis for potential therapeutic targeting. Future work will define the precise arginine methylation sites in JAK1 mediated by PRMT5 and systematically evaluate whether modulation of the PRMT5/JAK1/STAT3 pathway can mitigate SALI in CLP animal models.

## Funding

This study was supported by the Hangzhou Medical and Health Science and Technology Project, Project Number: B20254600.

## Ethics Statement

The study protocol involving the use of clinical samples was approved by the Medical Ethics Committee of No. 903 Hospital of PLA Joint Logistic Support Force (Approved No. 20240719/03/01/008). All procedures involving animal experiments were approved by the Animal Experimentation and Welfare Ethics Committee of Zhejiang Luoxi Medical Technology Co. Ltd., Hangzhou, China (Approved No. LX4825010601) and conducted in accordance with the Guide for the Care and Use of Laboratory Animals issued by the Ministry of Science and Technology of the People's Republic of China.

## Conflicts of Interest

The authors declare no conflicts of interest.

## Supporting information


**Data S1:** kjm270174‐sup‐0001‐DataS1.xlsx.

## Data Availability

The data that support the findings of this study are available from the corresponding author upon reasonable request.
